# Association between guilds of birds in the African-Western Palaearctic region and the tick species *Hyalomma rufipes,* one of the main vectors of Crimean-Congo hemorrhagic fever virus

**DOI:** 10.1016/j.onehlt.2021.100349

**Published:** 2021-11-11

**Authors:** Tove Hoffman, Laura G. Carra, Patrik Öhagen, Thord Fransson, Christos Barboutis, Dario Piacentini, Jordi Figuerola, Yosef Kiat, Alejandro Onrubia, Thomas G.T. Jaenson, Kenneth Nilsson, Åke Lundkvist, Björn Olsen

**Affiliations:** a Department of Medical Biochemistry and Microbiology, Zoonosis Science Center, Uppsala University, Husargatan 3, SE-751 23 Uppsala, Sweden; b Uppsala Clinical Research Center, Dag Hammarskjölds väg 38, SE-751 85 Uppsala, Sweden; c Department of Environmental Research and Monitoring, Swedish Museum of Natural History, Stockholm, Sweden; d Antikythira Bird Observatory, Hellenic Ornithological Society/BirdLife Greece, Athens, Greece; e Via Cesare Lippi 35, 400 26, Imola, BO, Italy; f Estación Biológica de Doñana, CSIC, Avda. Américo Vespucio s/n, 410 92 Sevilla, Spain; g CIBER Epidemiología y Salud Pública (CIBERESP), 280 29 Madrid, Spain; h Israeli Bird Ringing Center (IBRC), Israel Ornithological Center (IOC), Society for the Protection of Nature in Israel (SPNI), Tel-Aviv, Israel; i Migres Foundation, P.O. Box 152, 11380 Tarifa, Cadiz, Spain; j Department of Organismal Biology, Evolutionary Biology Centre, Uppsala University, Norbyvägen 18d, SE-752 36 Uppsala, Sweden; k Department of Medical Sciences, Section of Clinical Microbiology, Uppsala University, SE-751 85 Uppsala, Sweden; l Department of Medical Sciences, Zoonosis Science Center, Uppsala University, SE-751 85, Uppsala, Sweden

**Keywords:** African-Western Palaearctic, Migratory birds, Guilds, Ticks, *Hyalomma rufipes*

## Abstract

**Introduction:**

The ecology of the vertebrate host contributes to the geographical range expansion of ticks. In this study, we investigated which tick taxa that infest and are dispersed by birds along African-Western Palaearctic flyways during northward migration, and whether bird ecology was associated with tick taxa.

**Materials and methods:**

Ticks were collected from birds trapped at bird observatories in Spain, Italy, Greece, and Israel during the spring migration of 2014 and 2015, using mist nets. The tick-infested bird species were classified into guilds, using different combinations of the variables: migration distance, wintering region, foraging behaviour, and winter habitat. Ticks were molecularly determined to genus and species level by sequencing fragments of the 12S ribosomal DNA (rDNA) gene and by phylogenetic inference, using the Maximum Likelihood algorithm. Data were analysed using descriptive measures, graphs, Chi2 tests, the Tukey-Kramer test, and a parametric linear model (generalized linear model) in order to analyse and adjust for characteristics in the bird guilds and their relationship to collected tick taxa.

**Results:**

Most (84.2%) of the 10,209 trapped birds were long-distance migrants, of which 2.4% were infested by ticks. The most common tick species was *Hyalomma rufipes* (77.7%; 447/575), a known vector and reservoir of Crimean-Congo hemorrhagic fever virus. Bird guilds containing only long-distance migrants with wintering areas in Africa were associated with the tick species *H. rufipes* (*p* < 0.0001). Furthermore, bird winter habitat was associated with *H. rufipes* (*p* = 0.003); with bird species overwintering in open habitat (*p* = 0.014) and wetlands (*p* = 0.046) having significantly more *H. rufipes* as compared to birds with a winter habitat comprising forest and shrubs (*p* = 0.82).

**Conclusions:**

With climate change, the likelihood of establishment of permanent *Hyalomma* populations in central and northern Europe is increasing. Thus, surveillance programs for monitoring the risk of introduction and establishment of *H. rufipes* in the Western-Palaearctic should be established. Our study suggests that migratory bird species wintering in African open habitats and wetlands are good candidates for monitoring potential introduction.

## 1 Introduction

Habitat suitability, host abundance, and the ecology of the host are factors contributing to the geographical range expansion of ticks. Due to their migration patterns avian hosts are more likely to be involved in long-distance range expansion of ticks compared to terrestrial hosts. Each year billions of birds in the African-Palaearctic migration system (APMS) migrate between breeding grounds in the Palaearctic (including Europe, Asia North of the Himalayas, Northern Arabia, and Africa North of the Sahara) and wintering grounds (non-breeding area) in Africa [1], utilizing migration routes over the Mediterranean Sea. Together these routes connect the continents Europe, Asia, and Africa and constitute one of the World's largest bird migration systems, in which passerines are the most common [1,2]. Short-distance migratory bird species can perform the migration route in one flight, while long-distance migrants have to break their journeys at stopover sites (refuelling sites) along the migration route in order to complete the migration.

By their migration behaviour, birds facilitate transfer of ticks and pathogens between geographical sites. Many *Ixodes* and *Hyalomma* species are common ectoparasites of wild birds [3], especially of ground-feeding and ground-breeding bird species [4]. Tick species that spend long periods on their avian host can be transported over long distances. *Hyalomma marginatum* and *Hyalomma rufipes* are two-host ticks that moult from larva to nymph on the same host individual and can remain on the same host up to 26 days [5,6]. This behaviour enables dissemination of immature *H. marginatum* and *H. rufipes* from their normal distribution ranges in the Mediterranean region [7] and sub-Saharan Africa and regions around the Red Sea [8], respectively, to central and northern regions of Europe [[9], [10], [11]]. In the present study, we investigated which tick taxa that may be transported by birds migrating northward along African-Western Palaearctic (AWP) flyways. In addition, we analysed whether bird ecology was associated with tick taxa.

## 2 Materials and methods

### 2.1 Bird trapping and tick collection

**Fig. 1:**
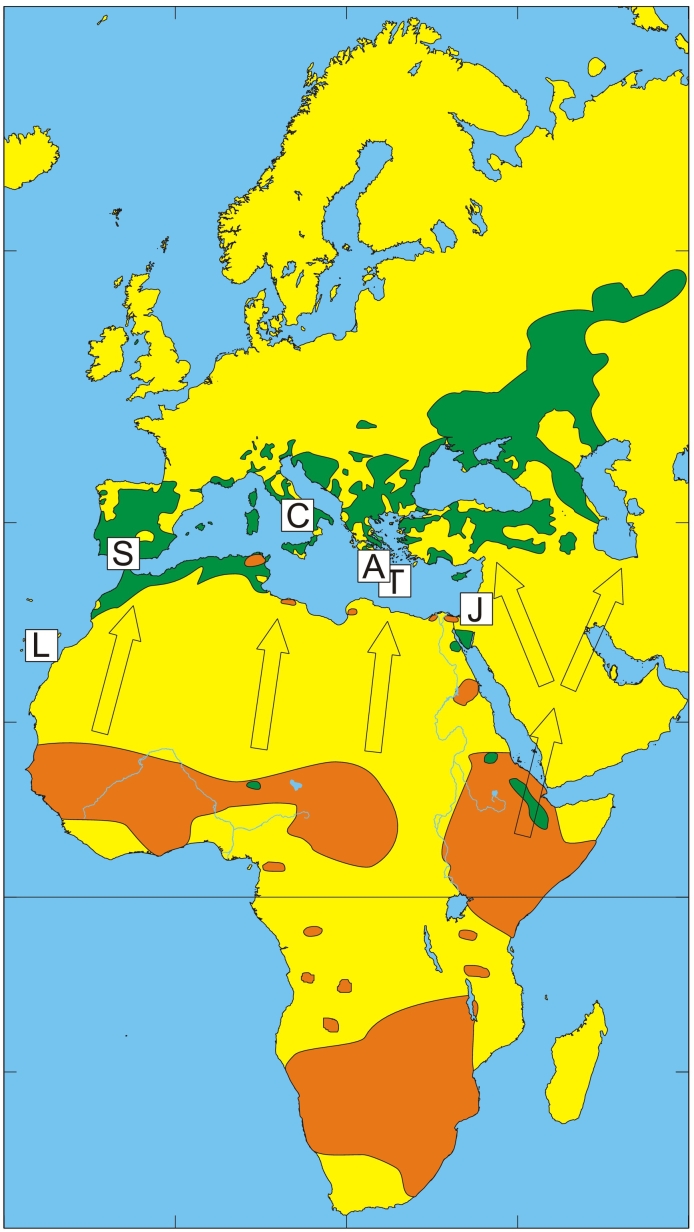
Map showing sites for trapping of birds and collection of infesting ticks (white squares). Known distribution areas of *Hyalomma rufipes* are indicated by orange and for *H. marginatum* by green, based on tick maps from the European Centre for Disease Prevention and Control (ECDC) and Walker et al. [12,13]. The black line indicates the Equator and arrows represent simplified northward migratory routes of birds in the African-Western Palaearctic. Trapping/collection sites are labelled: Canary Islands, Spain (L); continental Spain (S); Capri, Italy (C); Antikythira, Greece (A); Crete, Greece (T); and Jerusalem, Israel (J). (For interpretation of the references to colour in this figure legend, the reader is referred to the web version of this article.)

During ringing season, birds are regularly trapped by mist nets, ring-marked, measured, and weighed by ornithologists at bird observatories. In this study, birds trapped during the spring migration of 2014 and 2015 were included. Trapping took place at bird observatories in Spain, Italy, Greece, and Israel (Fig. 1): several sites in the provinces of Huelva and Sevilla, and on the Canary Islands (Spain; 37°30′N, 5°30′W; 37°33′N, 6°55′W; 28°9′N, 15°25′W); Capri (Italy; 40°33′N, 14° 15′E), the Anapodaris river, Crete (Greece; 34°59′N, 25°17′E); Antikythira (Greece; 35°51′N, 23°18′E); and Jerusalem and vicinity (Israel; 31°47′N, 35°13′E). Before release, each bird was visually inspected for ticks by ringers at the bird observatories by blowing apart the feathers. Collected ticks were stored in RNAlater™ Stabilization Solution (Invitrogen, ThermoFisher Scientific, MA, US) and refrigerator temperature until the end of the ringing season. Thereafter, ticks were stored at −80 °C. The aim was to investigate northbound migrating birds, i.e., birds trapped between March and June. Fourteen birds, with 32 infesting ticks, were excluded as they were trapped between July and October and could represent birds migrating southward. For this reason, ticks from the Spanish province Huelva and the Canary Islands were not included. The sampling approach of the present study, including trapping of birds with mist nets and visual inspection for ticks, may have resulted in sampling biases and an over- or under-representation of bird- and tick taxa.

### 2.2 Classification of bird species

Bird species infested by ticks were classified according to the variables: i) migration distance (long; medium; resident/short), ii) wintering region (South Europe; South Europe and North-West Africa; South Europe and Africa North of the Equator; Africa North of the Equator and South of the Sahel; Africa South of the Equator), iii) foraging behaviour (aerial; shrubs and trees; ground), and iv) winter habitat (wetland; open habitat; forest and shrubs) [[14], [15], [16], [17], [18], [19]]. Bird species were further classified into 11 guilds, groups of species with shared characteristics [20], because several tick-infested bird species were represented by only one individual (Table S1 in Appendix A). Guilds were created by using different combinations of the bird variables above. Most of the guilds were formed by long-distance migrants (8 out of 11), two by medium-distance migrants, and one by a resident/short-distance species. One guild had wintering regions in South Europe, one guild had wintering regions in South Europe and North-West Africa, two guilds had wintering regions in South Europe and Africa North of the Equator, four guilds had wintering regions in Africa North of the Equator and South of the Sahel, and three guilds had wintering regions in Africa South of the Equator. Regarding the foraging behaviour, most guilds comprised species that feed on the ground (5 out of 11) or in shrubs and trees (5 out of 11), whereas only one guild was formed by aerial feeding species. Forests and shrubs, was the winter habitat of seven guilds, while two wintered in wetlands and two in open habitats. The ecological characteristics and the bird species of each guild are presented in Table S2 in Appendix A.

### 2.3 Extraction of DNA

Ticks were individually surface-sterilized using absolute ethanol (Sigma-Aldrich, Merck, Darmstadt, Germany) and rinsed twice in sterile water before extraction of nucleic acids. Mechanical homogenization of individual ticks was conducted in a biosafety level 3 laboratory at 20 Hz for 5 min using a TissueLyser II (Qiagen, Hilden, Germany), a 5 mm stainless-steel bead (Qiagen, Hilden, Germany), and 200 μL TRIzol™ (Invitrogen, ThermoFisher Scientific, MA, USA). After homogenization, additionally 800 μL of TRIzol™ was added and the homogenate was transferred to a fresh microcentrifuge tube (Sarstedt, Nümbrecht, Germany). Homogenates were stored in −80 °C. The supernatant was collected after centrifugation at 14,000 x g for 30 s and used for isolation of nucleic acids. Ribonucleic acid (RNA) was extracted first using a manual phase separation technique with 0.2 volumes of chloroform (Sigma-Aldrich, Merck, Darmstadt, Germany). After removal of the aqueous phase, the deoxyribonucleic acid (DNA) was extracted using a back-extraction buffer (BEB) containing 3 M guanidine thiocyanate (Sigma-Aldrich, Merck, Darmstadt, Germany), 1 M Trizma base (Sigma-Aldrich, Merck, Darmstadt, Germany), 50 mM sodium citrate (Sigma-Aldrich, Merck, Darmstadt, Germany), and 250 mL of ultrapure diethyl pyrocarbonate (DEPC) treated water from Ambion (Invitrogen, ThermoFisher Scientific, MA, USA). In brief, 0.75 mL of BEB solution per 1 mL TRIzol™ used for the RNA extraction was added to the organic phase. The solution was mixed by inversion for 3 min and spun for 30 min at 12,000 x g at room temperature. The upper phase was transferred to a fresh 1.5 mL microcentrifuge tube and the DNA was subsequently concentrated and purified using the Nucleospin gDNA Clean up kit by Macherey-Nagel (PA, USA), with the minor modification of adding 1.5 parts of the binding buffer DB. The DNA was eluted in 50 μL Buffer DE and stored at −20 °C.

### 2.4 Tick identification

Based on previous data, a majority of the collected ticks were expected to be immature *H. marginatum* sensu lato (s.l.) ticks [21]. Morphological species determination of immature engorged *H. marginatum* s.l. ticks, including the species *H. marginatum* and *H. rufipes*, is difficult [22]. Identification of ticks to genus and species level was therefore performed using molecular methods. The mitochondrial 12S ribosomal DNA (rDNA) gene [23] was chosen as genetic marker as it has been proven to resolve relatively recent speciation events among ticks [[24], [25], [26]]. In short, the PCR mix of 25 μL consisted of 2.5 μL buffer (10×), 800 μM deoxynucleotide triphosphates (dNTPs) (Invitrogen, Thermo Fisher Scientific, MA, USA), 3.5 mM MgCl_2_ (AB, Thermo Fisher Scientific, MA, USA), 1 μM of each primer (T1B: 5′-AAA CTA GGA TTA GAT ACC CT-3′, T2A: 5′-AAT GAG AGC GAC GGG CGA TGT-3′; Invitrogen. Thermo Fisher Scientific, MA, USA), 0.5 U Platinum Taq (Invitrogen, Thermo Fisher Scientific, MA, USA), 2.5 μL template, and 9.4 μL sterile water (Thermo Fisher Scientific, MA, USA). The temperature profile was as follow: 95 °C for 5 min; 5 cycles of 95 °C for 15 s, 51 °C for 30 s, and 68 °C for 30 s; 25 cycles of 95 °C for 15 s, 52 °C for 30 s, and 70 °C for 30 s; followed by a final elongation step at 72 °C for 7 min. The amplicon size was 320 base pairs.

Amplicons were treated with Illustra ExoProStar 1-step (GE Healthcare, IL, USA) before being Sanger sequenced at Macrogen (Amsterdam, the Netherlands). Obtained sequences were trimmed and assembled in the CLC Main Workbench 7 (Qiagen, Aarhus, Denmark). Sequences were sorted according to genus based on BLAST results (www.ncbi.nlm.nih.gov/blast/Blast.cgi), using the R software. Sequences were then aligned using the Multiple Alignment using Fast Fourier Transform (MAFFT) algorithm (https://www.ebi.ac.uk/Tools/msa/mafft/) [27], with default settings. Partial 12S alignments were edited manually and reference sequences were retrieved from the National Center for Biotechnology Information (NCBI) GenBank database (https://www.ncbi.nlm.nih.gov/genbank/). Additionally, morphologically determined specimens of *H. marginatum*, *H. rufipes,* and *Hyalomma lusitanicum* were included as reference sequences. Phylogenies were built in the software Molecular Evolution Genetics Analysis (MEGA) version 7 [28], using the Maximum Likelihood (ML) and Neighbour-Joining (NJ) algorithms. For the ML analyses the following substitution models combined with the models of invariable sites (I) or gamma distribution (G) were chosen using model testing in MEGA7: T92 (Tamura 1992) + I (*Hyalomma*/*Ixodes* spp. alignment), T92 + G (*Haemaphysalis* spp. alignment), and GTR (General Time Reversible) + G (*Amblyomma* spp. alignment). A representative of the tick genus *Dermacentor* was used as outgroup [29]. For the NJ analyses, the Maximum Composite Likelihood model (default choice) was used. Gaps were treated using the complete deletion option. The bootstrap analysis was conducted with 1000 replicates. Ticks were grouped according to their position in the ML phylogenies. The 12S rDNA gene tick sequences in this study can be provided upon request.

### 2.5 Statistical analyses

In order to be able to analyse and adjust for characteristics in the ecology of the bird species and in the characteristics between the bird species and that of the tick taxon, the data were analysed using descriptive measures, graphs, Chi2 tests, a parametric linear model (generalized linear model [GLM]), and the Tukey-Kramer adjustment for multiple comparisons test. Chi2 tests were applied to determine whether guilds differed in their degree of tick infestation and whether there was an association between avian guild and tick taxon. The GLM was applied to determine whether there was an association between bird characteristics and tick taxa. We did not adjust for multiplicity at the study level, i.e., all tests were at 5%-level. However, all tests comparing more than two groups were adjusted for multiplicity issues. The Tukey-Kramer adjustment for multiple comparisons test was performed at test level and used for separation of means, i.e., comparing bird characteristic variables. All results should therefore be viewed as exploratory. GLM was preferred over non-parametric tests since GLM is better suited for adjusting for several covariates. For some of the GLM tests a Wilcoxon test was used as a sensitivity analysis. Statistical analyses were conducted in SAS (Statistical Analysis Software) version 9.4 (SAS Institute Inc., NC, USA**)**. *P* values < 0.05 were considered statistically significant.

## 3 Results

### 3.1 Descriptive statistics of birds and ticks

During the spring migration of 2014 and 2015, a total of 10,209 birds of 27 different species were trapped, ring-marked, and investigated for ticks. Long-distance migrants were most common (84.2%; 8594/10,209) (Table 1). The most commonly trapped bird species was the garden warbler (*Sylvia borin*) (16.4%; 1670/10,209) (Table S1 in Appendix A). In total, 2.4% (244/10,209) of the birds were infested by ticks, of which the whinchat (*Saxicola rubetra*) (19.3%; 47/244) and the whitethroat (*Sylvia communis*) (14.3%; 35/244) were the most common species (Table S3 in Appendix A). Most of the tick-infested birds were trapped on Capri in Italy and Antikythira in Greece (Table S1 in Appendix A) and classified as long-distance migrants (98.0%; 239/244) (Table 1). Most bird individuals were in the guilds C, I, and J, while guilds B and D were represented by one bird individual each (Table 2).

**Table 1:** Number of trapped and tick-infested birds per migration pattern.

Migration pattern	Trapped birds (%)	Tick-infested birds (%)
Resident/Short distance	27 (0.3)	1 (0.4)
Medium distance	1588 (15.6)	4 (1.6)
Long distance	8594 (84.2)	239 (98.0)
**Total**	**10,209 (100)**	**244 (2.4)**

**Table 2:** Number of tick-infested birds and ticks per avian guild.

Tick-infested birds		Infesting ticks		
Guild	Sum (%)	Median	Max	Sum (%)
A	18 (7.4)	2	22	61 (10.6)
B	1 (0.4)	1	1	1 (0.2)
C	61 (25.0)	2	17	184 (32.0)
D	1 (0.4)	2	2	2 (0.4)
E	6 (2.5)	1	2	8 (1.4)
F	21 (8.6)	1	6	36 (6.3)
G	2 (0.8)	2	3	4 (0.7)
H	2 (0.8)	1	1	2 (0.4)
I	49 (20.1)	1	10	91 (15.8)
J	81 (33.2)	2	11	182 (31.7)
K	2 (0.8)	2	2	4 (0.7)
**Total**	**244**	**2**	**22**	**575 (100)**

A total of 575 ticks were collected from the 244 birds, giving an average of 2.4 ticks per bird. *Saxicola rubetra* was the bird species with the highest number of ticks (Table S3 in Appendix A). The guilds A, C, F, I, and J had the highest number of ticks (Table 2). The collected ticks were found within 11 different groups (Table 3), based on their positioning in the phylogenetic trees (Fig. S1 in Appendix A) and sequence similarity, and represented four different Ixodidae genera: *Hyalomma* (83.8%), *Ixodes* (3.0%), *Amblyomma* (1.4%), and *Haemaphysalis* (0.3%). In total, 11.5% (66/575) of the ticks could not be molecularly determined due to absence of PCR amplicon. The most common tick species were *H. rufipes* and *H. marginatum* (Table 3).

**Table 3:** Identified tick groups and their descriptive statistics.

Tick group	Tick family/genus/sp.	Median	Mean	Max	Sum (%)
1	Ixodidae*/Ixodes/Ixodes frontalis-*like	0	0.03	1	6 (1.0)
2	Ixodidae*/Ixodes/Ixodes ricinus-*like	0	0.004	1	1 (0.2)
3	Ixodidae*/Ixodes/Ixodes* sp.	0	0.004	1	1 (0.2)
4	Ixodidae*/Ixodes/Ixodes* sp.	0	0.04	2	9 (1.6)
5	Ixodidae*/Hyalomma/Hyalomma marginatum*	0	0.1	6	24 (4.2)
6	Ixodidae*/Hyalomma/Hyalomma rufipes*	1	1.8	20	447 (77.7)
7	Ixodidae*/Hyalomma/Hyalomma sp.*	0	0.05	4	11 (1.9)
8	Ixodidae*/Haemaphysalis/Haemaphysalis* sp.	0	0.008	2	2 (0.4)
9	Ixodidae*/Amblyomma/Amblyomma* sp.	0	0.02	5	5 (0.9)
10	Ixodidae*/Amblyomma/Amblyomma* sp.	0	0.01	1	3 (0.5)
11	Unknown (ND)	0	0.3	9	66 (11.5)
**Total**		**2**	**2.4**	**22**	**575**

ND, No data; sp., species.

The guilds differed in their degree of tick infestation (*p*=0.00014, Chi2 test). Guilds A and C had more ticks than expected while guilds F and I had less ticks than expected (Table 4). The number of ticks per tick taxa for each guild is presented in Table 5.

**Table 4:** Observed and expected numbers of ticks per bird guild.

Guild	Proportion of total number of birds	Observed number of ticks	Expected number of ticks
A	0.07	61	42.4
B	0.004	1	2.4
C	0.3	184	143.8
D	0.004	2	2.4
E	0.02	8	14.1
F	0.09	36	49.5
G	0.008	4	4.7
H	0.008	2	4.7
I	0.2	91	115.5
J	0.3	182	190.9
K	0.008	4	4.7

**Table 5:** Associations between bird guilds and tick groups.

Bird guild	B^a^	H	K	A	C	D^a^	E	F	G	I	J		
Migration distance	R/S	M		L									
Tick group/Taxa (Distribution area/Zoogeographic region)		Number of ticks (%)										Total	P value^b^
1. *Ixodes frontalis-*like (*I. frontalis* is present in the PA [Europe, western Asia, NA]) [30]		2 (33.3)	1 (16.7)					1 (16.7)		1 (16.7)	1 (16.7)	**6** (1.0)	
2. *Ixodes ricinus-*like (*I. ricinus* is widely distributed in the WPA) [31]											1 (100)	**1** (0.2)	
3. *Ixodes* sp.								1 (100)				**1** (0.2)	
4. *Ixodes* sp.			1 (11.1)					1 (11.1)		7 (77.8)		**9** (1.6)	
5. *Hyalomma marginatum* (PA [Southern Europe, parts of Asia, NA]) [7]	1 (4.2)		2 (8.3)		6 (25.0)						15 (62.5)	**24** (4.2)	
6. *Hyalomma rufipes* (AT [sub-Saharan Africa, regions around the Red Sea]) [8]				54 (12.1)	151 (33.8)	2 (0.4)	5 (1.1)	19 (4.3)	4 (0.9)	65 (14.5)	147 (32.9)	**447** (77.7)	<0.0001
7. *Hyalomma* sp.					11 (100)							**11** (1.9)	
8. *Haemaphysalis* sp.					2 (100)							**2** (0.3)	
9. *Amblyomma* sp.					5 (100)							**5** (0.9)	
10. *Amblyomma* sp.					1 (33.3)			2 (66.7)				**3** (0.5)	
11. Unknown (ND)				7 (10.6)	8 (12.1)		3 (4.5)	12 (18.2)		18 (27.3)	18 (27.3)	**66** (11.5)	<0.0001
**Infesting ticks** (%)	**1** (0.2)	**2** (0.3)	**4** (0.7)	**61** (10.6)	**184** (32.0)	**2** (0.3)	**8** (1.4)	**36** (6.3)	**4** (0.7)	**91** (15.8)	**182** (31.7)	**575** (100)	<0.0001
**Tick-infested birds** (%)	**1** (0.4)	**2** (0.8)	**2** (0.8)	**18** (7.4)	**61** (25.0)	**1** (0.4)	**6** (2.5)	**21** (8.6)	**2** (0.8)	**49** (20.1)	**81** (33.2)	**244** (100)	
**Trapped birds** (%)	**27** (0.3)	**1313** (12.9)	**275** (2.7)	**527** (5.2)	**853** (8.4)	**26** (0.3)	**501** (4.9)	**1254** (12.3)	**738** (7.2)	**3278** (32.1)	**1417** (13.9)	**10,209** (100)	

a Guild represented by a single bird individual. ^b^ Statistical analyses were conducted in SAS, using a Chi2 test. The p value is for the test that all guilds are equal in terms of attracting a tick group and was considered significant when <0.05. R/S, Resident/Short; M, Medium; L, Long; PA, Palaearctic (Europe, North Africa, Asia, and North of the Himalayas); WPA, Western Palaearctic (Europe, North Africa, northern and central parts of the Arabian Peninsula, and part of temperate Asia); NA, Northern Africa; AT, Afrotropical (Africa south of the Sahara and the southwest tip of Arabia); ND, No data.

### 3.2 Guild association with tick taxon

There was an association between bird guilds and tick taxon (*p* < 0.0001, Chi2 test) (Table 5). Guilds containing only long-distance migrants with wintering areas in Africa were associated with the species *H. rufipes* (p < 0.0001, Chi2 test).

### 3.3 Habitat association with tick species

**Fig. 2:**
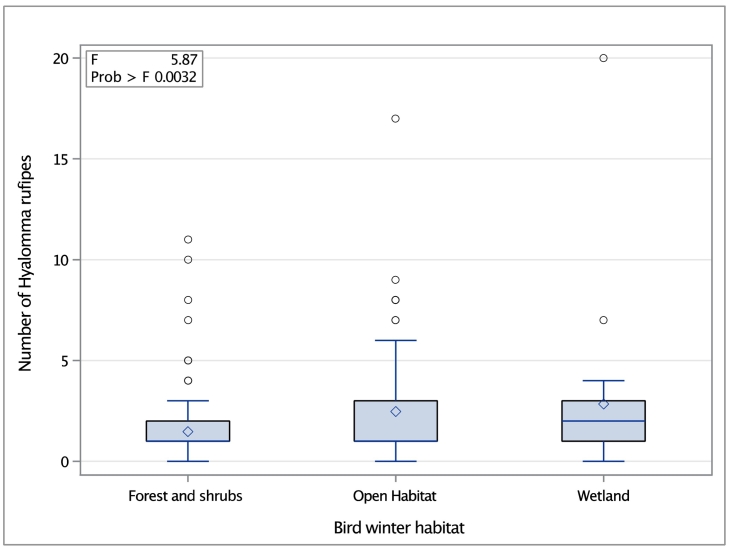
Box plot showing the distribution of *Hyalomma rufipes* ticks on birds with different winter habitats. The box plot shows that the winter habitat of the avian host had a significant effect on the number of infesting *H. rufipes* (*p* = 0.0032, generalized linear model). Line indicates median, diamond mean, and circles outliers.

The winter habitat of the avian host was found to be associated with the tick species *H. rufipes* (p = 0.0032, GLM) (Fig. 2). Furthermore, birds overwintering in an open habitat (*p* = 0.014, Tukey-Kramer test) and in wetlands (*p* = 0.046, Tukey-Kramer test) were significantly more heavily infested by *H. rufipes* than bird species with a winter habitat comprising forest and shrubs (*p* = 0.82, Tukey-Kramer test). The number of tick-infested avian individuals according to their foraging behaviour and winter habitat is presented in Table S4 in Appendix A.

## 4 Discussion

Birds within the APMS make a rapid return from their wintering areas to their breeding areas during the spring migration [32]. Birds that have depleted their fuel reserves after crossing geographical hurdles such as the Saharan Desert and the Mediterranean Sea must pause their journey at stopover sites to refuel and rest. In this study, predominantly long-distance migratory birds were trapped (84.2%; 8594/10,209) and infested by ticks (98.0%; 239/244). Most of the tick-infested birds were trapped on Antikythira in Greece and Capri in Italy, which are two of the first stopover sites in the Mediterranean Sea for birds flying northwards from wintering regions in Africa. *H. rufipes* (77.7%) and *H. marginatum* (4.2%) were the most common tick species. *H. rufipes* and *H. marginatum* are known to transmit pathogens of both medical and veterinary importance, including Crimean-Congo hemorrhagic fever virus (CCHFV), *Rickettsia conorii*, and *Anaplasma marginale* [7,8,33]*.* Several other pathogens, such as *Babesia occultans* and *Theileria annulata,* have also been associated with these tick species [7,8]. CCHFV causes severe and fatal hemorrhagic fever in humans and is endemic in Africa, South-Eastern Europe (the Balkans being the known western limit), the Middle East, and Asia [34]. In Africa and Europe, the main vectors of CCHFV are *H. rufipes* and *H. marginatum*, respectively [34,35]. They also serve as reservoirs for the virus. During the past decades, the incidence of Crimean-Congo hemorrhagic fever (CCHF) has increased in endemic areas and the virus has emerged in new regions. Recently, autochthonous cases of CCHF were detected in South-Western Europe, in Spain [36]. The causative agent was a virus related to the African 3 lineage of CCHFV with a high similarity to the West-African Mauritania strain ArD39554 from a *H. rufipes* (GenBank accession number: DQ211641) [36], indicating importation of the virus from Northern Africa possibly via infected ticks infesting northward migrating birds coming from Africa [37]. Detection of genetic material of CCHFV, likely of African origin, in adult *H. lusitanicum* ticks from red deer (*Cervus elaphus*) in Spain [38] further support an introduction from Africa as well as suggest a possible circulation of the virus in Spain. Genetic material of CCHFV [39], *Rickettsia aeschlimannii* [21]*,* and Alkhurma hemorrhagic fever virus [40] have previously been detected in *H. marginatum* s.l. ticks infesting northbound migratory birds trapped in the study area.

The tick species *H. rufipes* [8,41], which is an Afrotropical species distributed in the drier parts of Africa and along the Red Sea cost on the Arabian Peninsula [22,41], was found exclusively on long-distance AWP migratory birds with wintering regions in Africa (*p* < 0.0001) (Table 5). The seasonal movements of the low air pressure belt at the Equator in Africa make areas just south of the Sahara, including the Sahel region, very dry during the northern winter until the departure of Palaearctic birds in the spring [42]. Many Palaearctic birds therefore spend their non-breeding season in African regions with low rainfall, yet in an ecosystem that provides both food and shelter. In this study, bird winter habitat was found to be associated with the dry open country tick species *H. rufipes* [8,43] (*p* = 0.0032); with birds in an open winter habitat having significantly more *H. rufipes* (*p* = 0.014) than birds in a winter habitat comprising forest and shrubs (*p* = 0.82). Furthermore, birds with a wetland winter habitat were found to have significantly more *H. rufipes* (*p* = 0.046) than birds in a forest and shrubs habitat (p = 0.82), possibly due to foraging in *H. rufipes* habitat and/or being less selective of habitat during the migration.

Considering that more than a billion birds within the APMS might cross the Mediterranean Sea during the yearly spring migration [1], millions of infesting ticks are likely to be transported from the African to the European continent each year. The limiting factor for the geographical distribution of avian-associated ticks is not only their dissemination ability but presumably even more the ecological and climatological suitability of the area where these ticks detach. An important geographical determinant for ticks is the climate. Southern Europe and North Africa [7,8] are the northern distribution limits of *H. marginatum* and *H. rufipes* (Fig. 1)*,* respectively**,** which is likely to change in the next few decades due to global warming [44]. Adult *H. marginatum* and *H. rufipes* have recently been reported from Central and Northern Europe [[45], [46], [47], [48], [49], [50], [51]], which is outside their normal distribution ranges [7,8]. The risk that these tick species are expanding their geographic distribution areas northwards in Europe should not be neglected, since it might increase the likelihood that the pathogens carried by these tick species, such as CCHFV, will become endemic and enzootic in new areas. Detection of adult specimens outside hitherto endemic areas is likely a result of avian-associated introduction of immatures and warm weather conditions allowing moulting into the adult stage. Thus, with a warmer climate together with a large number of available hosts for the adult ticks the likelihood of completion of the life cycle, human exposure to locally acquired *Hyalomma* ticks [52], and establishment of permanent *Hyalomma* populations in central and northern regions of Europe will increase. Based on our results, it is likely that long-distance migrants, either wintering or stopping over in arid areas, are involved in the transportation of *H. rufipes* to countries at higher latitudes, like Sweden [45]. Such transportation is possible due to the very long feeding time of immature *H. rufipes* ticks on the same host [5] and can last for several weeks or may take just a few days depending on the bird species. This is supported by ringing recoveries of willow warblers (*Phylloscopus trochilus*), a long-distance migrant species with wintering areas in sub-Saharan Africa, indicating that they regularly migrate from the Mediterranean Sea to Sweden during the spring migration in less than 14 days (Bird Ringing Center, Swedish Museum of Natural History).

In conclusion, this study highlights the need to establish surveillance programs for monitoring the risk of introduction and establishment of *H. rufipes* in the Western Palaearctic. Our study suggests that migratory bird species wintering in African open habitats and wetlands are good candidates for monitoring potential introduction.

## Ethical consideration

Ticks were collected from birds in connection with ordinary scientific bird ringing activities and carried out by trained and licensed ringers, under the following permits and licenses: Spain) 660117 and 180007 issued by the Ministerio de Agricultura, Pesca y Alimentación, and 66042 issued by Consejeria de Agricultura, Ganadería, Pesca y Desarrollo Sostenible; Italy) 59019 issued by L'Istituto Superiore per la Protezione e Ricerca dell'Ambientale (ISPRA); Greece) ΑΔΑ:ΒΛ9Σ0-Γ3Α, ΑΔΑ:Β4ΩΖ0-Ν6Χ, ΑΔΑ:ΩΗΛΔ465ΦΘΗ-31Γ, and ΑΔΑ: ΩΧΒΠ465ΦΘΗ-ΒΧΥ issued by the Hellenic Ministry of Environment and Energy; and Israel) A258 issued by the Israel Nature and Parks Authority.

## Availability of data and materials

The data sets used and analysed during the current study are available from the corresponding author on reasonable request.

## Funding

This study was supported by ALF grants from the 10.13039/501100009230Uppsala County Council, the 10.13039/501100000780European Union‘s Horizon 2020 research innovation program under the grant no. 874735 (VEO), and the Ax:son Johnson Foundation.

## Authors´ contributions

TH organized the project, performed the molecular species determination, analysed and interpreted the data, and wrote the original draft of the manuscript. LGC assisted in the molecular determination of the ticks. PÖ performed the statistical analyses and supervised. TF classified the birds and supervised. CB, JF, YK, AO, and DP performed the trapping of the birds and collected the ticks. TGTJ and KN supervised. ÅL supervised and funded the project. BO supervised, organized, and funded the project. All authors have read and commented on the manuscript.

## Declaration of Competing Interest

The authors declare that they have no competing interests.

## Appendix A Supplementary data

Supplementary material Image 1
