# Supplementary material for: Association between guilds of birds in the African-Western Palaearctic region and the tick species Hyalomma rufipes, one of the main vectors of Crimean-Congo hemorrhagic fever virus
Source: One Health. 2021 Nov 11;13:100349. doi: 10.1016/j.onehlt.2021.100349 (PMC8605260; doi:10.1016/j.onehlt.2021.100349)
Supplement: Supplementary file 1 — Supplementary material [file mmc1.pdf]

Supplementary data

Table S1. Number of trapped and tick-infested birds per bird species and collection site.

		**Nb of trapped tick-infested birds**							**Nb of trapped birds**							
**Bird species**	**Guild**	**Greece (Antikythira)**	**Greece (Crete)**	**Italy (Capri)**	**Israel**	**Spain**	**Sum**	**%**	**Greece (Antikythira)**	**Greece (Crete)**	**Italy (Capri)**	**Israel**	**Spain**	**Sum**	**%**	**Tick-infested birds (%)**
Acrocephalus schoenobaenus	A	13	1	1	2		17	6.97	35	18	20	40	5	118	1.16	14.41
Acrocephalus scirpaceus	A					1	1	0.41	21	15		301	72	409	4.01	0.24
Anthus trivialis	C	4		2			6	2.46	103	8	37	58	3	209	2.05	2.87
Caprimulgus europaeus	E	1					1	0.41	13	2	18			33	0.32	3.03
Charadrius alexandrinus	B					1	1	0.41					27	27	0.26	3.70
Erithacus rubecula	K	1					1	0.41	6		116	1	130	253	2.48	0.40
Falco naumanni	C					1	1	0.41					40	40	0.39	2.50
Ficedula albicollis	I	3	1	2			6	2.46	111	7	190	1		309	3.03	1.94
Ficedula hypoleuca	F	10	1	10			21	8.61	332	4	723		195	1254	12.28	1.67
Hippolais icterina	I	3		2			5	2.05	86	1	294			381	3.73	1.31
Lanius senator	J	14	2	1		2	19	7.79	77	9	13	2	10	111	1.09	17.12
Luscinia megarhynchos	J	3	2	1		1	7	2.87	73	38	56	6	66	239	2.34	2.93
Monticola saxatilis	C			1			1	0.41	0		1			1	0.01	100
Motacilla flava	D		1				1	0.41	14	6			6	26	0.25	3.85
Muscicapa striata	E	4		1			5	2.05	199	33	170	1	65	468	4.58	1.07
Oenanthe oenanthe	C			5			5	2.05	1	1	58			60	0.59	8.33
Oriolus oriolus	I	14	1	4			19	7.79	233	9	102			344	3.37	5.52
Phoenicurus phoenicurus	J	9		8	1	1	19	7.79	85		103	3	30	221	2.16	8.60
Phylloscopus collybita	H	2					2	0.82	29	1	36	58	1189	1313	12.86	0.15
Phylloscopus sibilatrix	I	5		10			15	6.15	126	13	434	1		574	5.62	2.61
Saxicola rubetra	C	6		41			47	19.26	60	10	441			511	5.01	9.20
Streptopelia turtur	J		1				1	0.41	67	7	5	1		80	0.78	1.25
Sylvia atricapilla	G	2					2	0.82	130	2	132	229	245	738	7.23	0.27
Sylvia borin	I	4					4	1.64	713	15	811	4	127	1670	16.36	0.24
Sylvia communis	J	11	1	23			35	14.34	86	34	528	17	101	766	7.50	4.57
Turdus philomelos	K	1					1	0.41	5			1	16	22	0.22	4.55
Upupa epops	C	1					1	0.41	23		8		1	32	0.31	3.13
**Total**		**111**	**11**	**112**	**3**	**7**	**244**		**2628**	**233**	**4296**	**724**	**2328**	**10,209**		**2.39**
%		45.49	4.51	45.90	1.23	2.87			25.74	2.28	42.08	7.09	22.80			

Table S2. Guilds of tick-infested bird species. Based on information in the Handbook of the birds of Europe, the Middle East and North Africa [13-18].

	Bird characteristics				
**Guild**	**Migration distance**	**Wintering region**	**Foraging behaviour**	**Winter habitat**	**Bird species (Common name)**
A	Long	Africa North of the Equator and South of the Sahel	Shrubs and trees	Wetland	*Acrocephalus schoenobaenus* (Sedge warbler)<br>*Acrocephalus scirpaceus* (Eurasian reed warbler)
B	Resident/short	South Europe	Ground	Wetland	*Charadrius alexandrinus* (Kentish plover)
C	Long	Africa North of the Equator and South of the Sahel	Ground	Open habitat	*Anthus trivialis* (Tree pipit)<br>*Falco naumanni* (Lesser kestrel)<br>*Monticola saxatilis* (Rufous-tailed rock thrush)<br>*Oenanthe oenanthe* (Wheatear)<br>*Saxicola rubetra* (Whinchat)<br>*Upupa epops* (Eurasian hoopoe)
D	Long	Africa South of the Equator	Ground	Open habitat	*Motacilla flava* (Yellow wagtail)
E	Long	Africa South of the Equator	Arial	Forest and shrubs	*Caprimulgus europaeus* (European nightjar)<br>*Muscicapa striata* (Spotted flycatcher)
F	Long	Africa North of the Equator and South of the Sahel	Shrubs and trees	Forest and shrubs	*Ficedula hypoleuca* (European pied flycatcher)
G	Long	South Europe and Africa North of the Equator	Shrubs and trees	Forest and shrubs	*Sylvia atricapilla* (Blackcap)
H	Medium	South Europe and Africa North of the Equator	Shrubs and trees	Forest and shrubs	*Phylloscopus collybita* (Chiffchaff)
I	Long,	Africa South of the Equator	Shrubs and trees	Forest and shrubs	*Ficedula albicollis* (Collared flycatcher)<br>*Hippolais icterina* (Icterine warbler)<br>*Oriolus oriolus* (Eurasian golden oriole)<br>*Phylloscopus sibilatrix* (Wood warbler)<br>*Sylvia borin* (Garden warbler)
J	Long	Africa North of the Equator and South of the Sahel	Ground	Forest and shrubs	*Lanius senator* (Woodchat shrike)<br>*Luscinia megarhynchos* (Nightingale)<br>*Phoenicurus phoenicurus* (Common redstart)<br>*Streptopelia turtur* (Turtle dove)<br>*Sylvia communis* (Whitethroat)
K	Medium	South Europe and North-West Africa	Ground	Forest and shrubs	*Erithacus rubecula (*European robin)<br>*Turdus philomelos* (Song thrush)

Table S3. Number of collected ticks per bird species and tick group.

Bird species	Guild	Nb of ticks	Nb of tick-infested birds	Nb of ticks/bird	Tick group: *Ixodes frontalis*-like (1)	*Ixodes ricinus*-like (2)	*Ixodes* sp. (3)	*Ixodes* sp. (4)	*Hyalomma marginatum* (5)	*Hyalomma rufipes* (6)	*Hyalomma* sp. (7)	*Haemaphysalis* sp. (8)	*Amblyomma* sp. (9)	*Amblyomma* sp. (10)	ND (11)
Acrocephalus schoenobaenus	A	60	17	3.53						53					7
Acrocephalus scirpaceus	A	1	1	1.00						1					
Anthus trivialis	C	19	6	3.17						4	3		5	1	6
Caprimulgus europaeus	E	1	1	1.00						1					
Charadrius alexandrinus	B	1	1	1.00					1						
Erithacus rubecula	K	2	1	2.00					2						
Falco naumanni	C	2	1	2.00								2			
Ficedula albicollis	I	9	6	1.50						8					1
Ficedula hypoleuca	F	35	21	1.67	1		1	1		19				2	11
Hippolais icterina	I	7	5	1.40	1			1		5					
Lanius senator	J	50	19	2.63					2	45					3
Luscinia megarhynchos	J	19	7	2.71					5	14					
Monticola saxatilis	C	1	1	1.00						1					
Motacilla flava	D	2	1	2.00						2					
Muscicapa striata	E	7	5	1.40						4					3
Oenanthe oenanthe	C	28	5	5.60					6	21					1
Oriolus oriolus	I	40	19	2.11				6		30					4
Phoenicurus phoenicurus	J	32	19	1.68					2	29					1
Phylloscopus collybita	H	2	2	1.00	2										
Phylloscopus sibilatrix	I	30	15	2.00						21					9
Saxicola rubetra	C	132	47	2.81						123	8				1
Streptopelia turtur	J	3	1	3.00						3					
Sylvia atricapilla	G	4	2	2.00						4					
Sylvia borin	I	5	4	1.25						1					4
Sylvia communis	J	79	35	2.26	1	1			6	56					15
Turdus philomelos	K	2	1	2.00	1			1							
Upupa epops	C	2	1	2.00						2					
**Total**		**575**	**244**	**2.36**	**6**	**1**	**1**	**9**	**24**	**447**	**11**	**2**	**5**	**3**	**66**
%					1.04	0.17	0.17	1.57	4.17	77.74	1.91	0.35	0.87	0.52	11.48

sp., species; ND, No data.

Table S4. Number of avian individuals with ticks according to their foraging behaviour and winter habitat.

	**Foraging behaviour**			
**Winter habitat**	Arial	Shrubs and trees	Ground	**Total**
Wetland	0	18	1	19
Open habitat	0	0	62	62
Forest and shrubs	6	74	83	163
**Total**	**6**	**92**	**146**	**244**

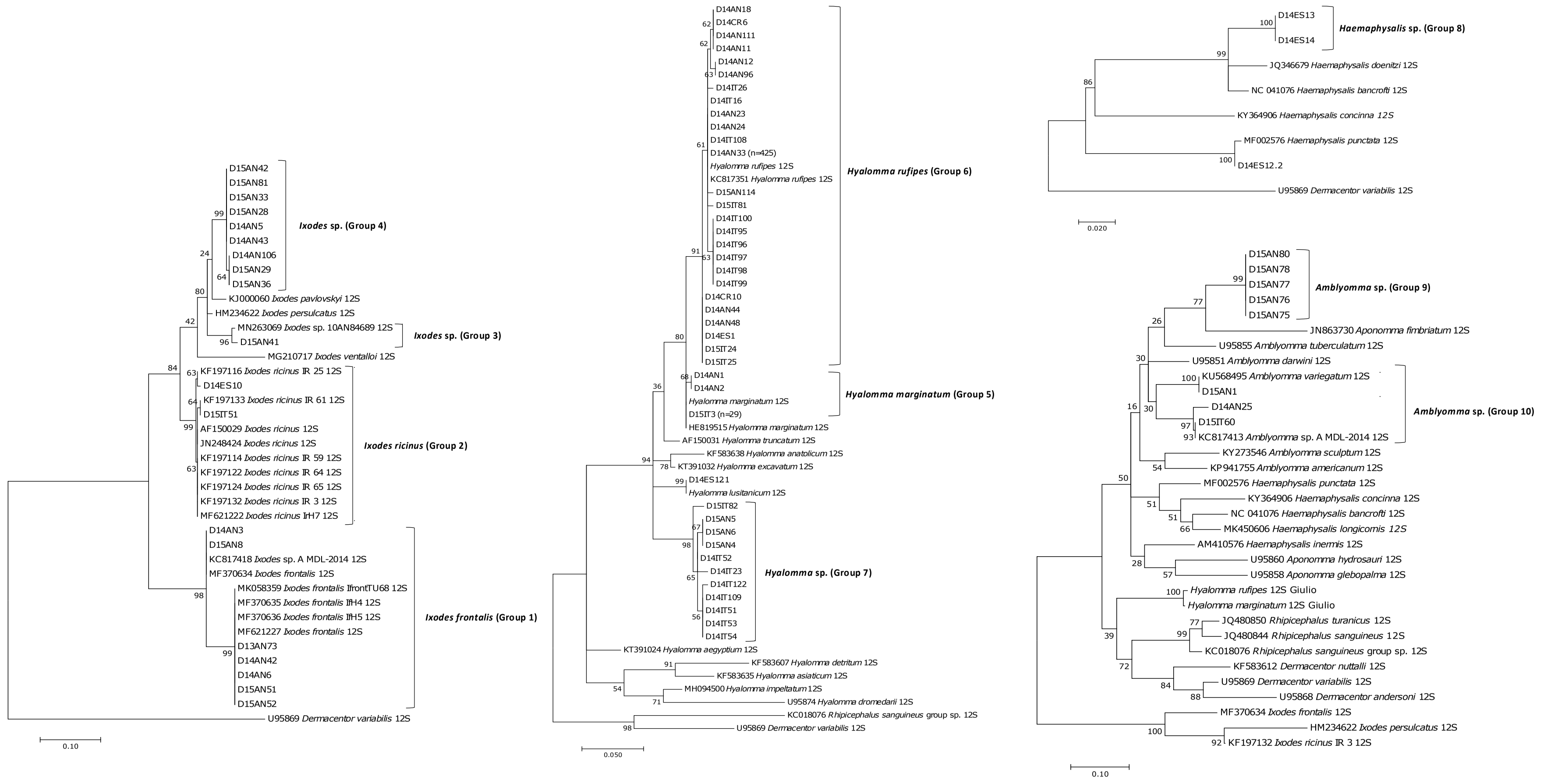

Figure S1. Phylogenetic trees, including all collected ticks, showing the groups (in bold) of the investigated partial 12S rDNA tick sequences. The phylogenies were inferred using the Maximum Likelihood algorithm and the substitution models T92 + I (*Hyalomma*/*Ixodes* spp.), T92 + G (*Haemaphysalis* spp.), and GTR + G (*Amblyomma* spp.) in the software MEGA7. GenBank accession numbers of reference sequences and bootstrap values are presented in the trees. The scale bars indicate the number of substitutions per site.
